# Pyorrhea and Its Constitutional Effects

**Published:** 1914-07

**Authors:** William Crenshaw

**Affiliations:** Atlanta, Ga.; Dean Atlanta Dental College


### PYORRHEA AND ITS CONSTITUTIONAL EFFECTS.

By William Crenshaw, D. D. S., Dean Atlanta Dental College.

Alveolar dental pyorrhea, commonly called Rigg's Disease, was first described by Pierre Fauchard, a French pathologist, two hundred years ago. Fauchard did not suggest a treatment, and like Crawford Long in the discovery of ether anaesthesia, did not realize then what his discovery would lead to, nor dream what direful consequences the disease must in the future unfold to its credit.

I say this, gentlemen, because undoubtedly alveolar dental pyorrhea is undermining the health of thousands in its insidious progress. The medical profession whose province is not to treat pyorrhea, cannot be held responsible, because it lies at the door of the dental profession. Besides, until a study of the constitutional effects of the malady shall have settled into a more definite symptomatology, the medical profession cannot be held accountable for its effects.

What pyorrhea is, its course and its constitutional effects, I take it, are the facts that will interest a body of physicians.

In the beginning I feel that it is important to say that pyorrhea does not arise constitutionally nor systematically as the result of excessive uric acid elaboration. Pyorrhea is purely local in origin, and is itself the cause instead of the effect of pronounced constitutional disorders. How improbable it is to assume that a normal blood composition can generate bacteria of itself! But how rational and reasonable it is to believe that toxins absorbed from ulcerating pyorrhea pockets and pus swallowed with food, can and do poison the blood-stream.

Mechnikoff advanced the theory of phagocytic immunity. He showed, I believe correctly, how opsonins prepare the white blood corpuscles for engulfing and digesting bacteria, and thereby neutralizing bacterial invasion of the blood; and how the blood does not tolerate bacteria, except in those conditions similar to pyorrhea, in which the opsonic attribute of the blood is overwhelmed, overpowered and broken down, and the disease forces gain ascendency over those of health.

Besides, blood examination from cases of pyorrhea show by the absence of bacteria that it is erroneous to ascribe the disease to bacteria in the blood, or to constitutional causes, except as they arise secondarily from the primary infection and toxemia generated in the pyorrheal ulcers.

Is it reasonable, then, to ascribe to an otherwise pure blood-stream the establishment of suppurative ulcers in the mouth, around the teeth or elsewhere, without first showing the presence in the blood of infective and toxic material resulting from local pyorrhea in the mouth, especially when pus is going with the food through the alimentary tract, through the digestive and assimilative processes?

The mouth is as perfect bacterial incubator as can be devised. Here we have the laboratory PAR EXCELLENCE for the production of pathogenic microorganisms, from whence toxic material is generated, swallowed and absorbed. Thus it is easier to understand how one should die rather than how one should recover from advanced Rigg's disease.

Pyorrhea originates initially from the deposition of salivary calculus beneath the free edge of the gum. This deposit increases and hardens. Its first effect is noticed in a slightly reddened gum margin. Slowly but steadily the redness changes into inflammation and into suppuration, and descends beneath the gum until the border of the alveolus and the ligament covering the root is reached. Here a progressive necrosis sets in of both the alveolus and dental ligament, rather than of the gum tissue. In the destruction of these two tissues results the pus that exudes from around the teeth.

In the bacteriological examination of pyorrhea-pus are found staphylococci, streptococci and pneumococci.

"Pyorrhea" means, literally, flow of pus; while the word "alveolaris" signifies relation to the alveolus. The disease proceeds slowly, stealthily, insidiously; and rarely causes pain or recognition of its presence until the ulcerative pockets have deepened and enveloped one or more of the roots of the teeth.

Kept up through a series of years the pus absorption as it is swallowed produces as first effect, derangement of the appetite and nutrition; pallor, languor, and weakening of the vital forces, falling off of weight, sleeplessness, rheumatism septic fever, or endocarditis may follow.

Well advanced into the latter stage, I have not known a case to recover, nor to live a great length of time after the fever set in.

One of the the first symptoms pointing to pyorrhea is excessive bleeding from hypertrophied gum-festoons between the teeth. This is a manifestation of the disease, which when it occurs, (and it does not occur invariably) points to pyorrhea. Another unmistakable symptom is the pyorrheal breath—a characteristic sickening, fetid breath that the patient himself does not recognize, but that everyone else does. A further manifestation of deep seated pyorrhea is the dark purplish gum over the affected teeth,—the thickened gums show at a glance, and do not attach to the teeth.

One other easily found pointer to pyorrhea is loose teeth. Usually it is most pronounced around the lower front teeth, which are in close proximity to the sublingual ducts, with abundant saliva secretion.

Probably eight-tenths of all pyorrhea originates inside the arches of the teeth, because the present day brush does not properly cleanse the inside of the teeth.

In the more rapidly advancing forms of this disease, and in persons of very robust constitutions, the disease removes the teeth, and thus automatically stops further poisoning of the system. But in the instances of weak constitutions, pyorrhea alveolaris goes through the grave systemic symptoms above described, and if left to its course may be expected to eventuate in a fatal termination.

619-21 Grant Building.

---

### REGULAR MEETING FULTON COUNTY MEDICAL SOCIETY, JULY 2ND, 1914.

By R. R. Daly, M. D., Atlanta, Ga.

Dr. W. B. Emory, in a talk on "The Last Word in Genito-Urinary Work," gave his impressions gained at the recent meeting of the A. M. A.

As to prostatectomy, the phthalein test might well be disregarded, because the operation is not contraindicated when one kidney is not well. Brash gave it as his opinion that the operation should be performed if not otherwise indicated. Geraghty said that one was on dangerous ground when operating with a diseased kidney.

McGowan, of Los Angeles, said that a tuberculous kidney did not get well of itself nor unde the care of the general practitioner. It needed surgical attention and that as early as possible.

Cabot, of Boston, said that there was no haemic infection of the kidney; the infection was lymphatic and that when one kidney is diseased, the other can not be infected by the passage of the organism along the ureters.

Lowe, of Cincinnati, told of packing the cavity in operation for diverticulum of the Bladder and thus giving a mass wherein dissection could be readily done after the supra-pubic section had been made.

Keyes, of New York, described a method of controlling haemorrhage after protatectomy. It consisted in making a purse string suture around the cavity whence the gland had been removed and drawing it tight from the outside. The suture was introduced from without by a needle similar to that one used in the external tying of varicocele.

Dr. Emory said he could find nothing new in the treatment of gonorrhoea. He did see the cystoscope used as an endoscope and treatment applied that way.

Returning to Atlanta, he was greatly interested to see that some of the men were doing Epididymotomy under cocaine at the office and allowing the patient to leave. He wondered if that was right and said that it was a distinct advance in the convenience of surgery if it were safe. One doesn't have to go to Atlantic City to find things in the lead as to surgery.

---

### REGULAR MEETING FULTON COUNTY MEDICAL SOCIETY, JULY 16, 1914.

Dr. Archibald Smith read a paper on "High Forceps and Caesarian Section,'.

Dr. O. H. Matthews said that when the true conjugate diameter of the pelvis is 8 1-2 c. m. or less, the application of high forceps can not be done successfully. There is invariably grave injury done to the mother or to the child or to both. If this measurement is known in advance of labor it indicates that Caesarian section should be done. Careful measurements should always be taken when possible so that the obstetrician is able to give advice and conduct the case properly. If for instance the condition is known before labor has begun and before forceps have been applied, the high section should be made. If, on the other hand, labor has commenced and there has been some injury done to the parts whereby sepsis may be started, the low section should be made and kept extra-peritoneal. Forceps should never be applied or attempts made to apply them to the floating head. Pituitrin has not yet done away with the need for forceps in his own opinion and experience.

Dr. McCord said he would prefer version to the use of forceps in some of the cases described where Caesarian section was not permitted.

Dr. Hardin said that the child is likely to die in the version cases and that section was indicated and should be insisted on. If taken in time, it can be done with greater safety than attends the use of forceps when the conjugate is small, and there is one good point about it: the vessels are under the operator's fingers in case of haemorrhage and he can control the bleeding.

The use of forceps involves the dilatation of the sphincter and this is the same wherever sphincters are handled in that there is tearing of tissue and danger of infection.

Dr. F. P. Calhoun reported a case of amblyopia due to infection of the sphenoidal sinus. The man first had slightly blurred vision in what had been normal sight. In a few days there were choked disc and almost total loss of vision. The history of the case was negative except that he used several handkerchiefs a day and had some post-nasal screatus. He was put on mixed treatment and rest. There were albumen, urea and casts. A polypus was discovered in the right naris and removed, then a skiagram was made by Derr and it was clearly shown that the sphenoidal sinus on that side was dull and filled with abnormal contents. The plate was exhibited at the Society. The midturbinate was removed and the sinus opened and curetted. Considerable pus was removed. There was prompt return of vision in the left eye, and slower improvement in the right, but both were normal now. From the time of examination to operation was about two weeks.

As to the avenue of infection leading from the sinus to the optic tract, Calhoun mentioned the work of a German investigator who has studied similar cases and said that it might be through the cavernus sinus or by means of dehiscence of bone along the wall of the sphenoidal sinus or by lymphatic means. The distance from one disc to another is short and may be well traversed by the organism passing along the optic nerve sheaths across the chiasm.

Dr. Lokey said that such cases point out the close relation of the sinuses to the optic tract and we are learning to look for it more and more.

Dr. Daly said that the presence of the polyp in the nose in connection with the amblyopia was significant in the beginning. In his work with the sinuses, he had found that the introduction of irritating substances into the sinuses had irritated the eyes and also that with the treatment of mild sinus difficulties by the introduction of gentle antiseptics, he had been able to relieve many ocular difficulties that had appeared stubborn before. It all went to show that a careful examination of the eye, no matter what condition was complained of, indicated also a careful examination of the nose. The separation of special work as to the eyes on the one side and nose and throat on the other was not practical.

Dr. J. S. Derr exhibited a skiagram of stone in the bladder of a four year old child. He had never seen or heard of one before. He also had a skiagram of stone in the kidney of a three year old child.

---

In surgical treatment when to remove a drain is almost as important a question as when to use one.—American Journal of Surgery.

---

Urinary leakage, even for a few weeks after ureterorrhaphy or ureteroanastomosis does not necessarily mean failure. It may cease spontaneously. American Journal of Surgery.

---

An acutely appearing almond-sized or larger swelling in the skin of the submetnal region, which the patient usually thinks is an inflamed gland, is not an uncommon winter occurrence, especially in women, from exposure to the cold. No treatment is necessary other than protection of the part from further chilling. The prominences of the cheeks may also be thus affected, but less frequently. Sudden swelling and redness of the nose, frightening the patient into a diagnoiss of erysipelas, is a less common "frost-bite" experience.—American Journal of Surgery.

